# Relationship Between Morphological Remodeling and Angiographic Types of Spontaneous Isolated Superior Mesenteric Artery Dissection After Conservative Management: Determinant Affecting Serial Radiologic Courses

**DOI:** 10.3389/fcvm.2022.945141

**Published:** 2022-07-07

**Authors:** Zihui Yuan, Shi Sheng, Yun You, Defu Li, Qi Wei, Kai Yan, Jian Wang

**Affiliations:** ^1^Department of Vascular Surgery, Union Hospital, Tongji Medical College, Huazhong University of Science and Technology, Wuhan, China; ^2^Department of Hepatobiliary and Gastrointestinal Surgery, Hankou Hospital, Wuhan, China

**Keywords:** spontaneous isolated superior mesenteric artery dissection, morphological remodeling, angiographic type, conservative management, false lumen enhancement, ulcer-like projection

## Abstract

**Objective:**

To monitor the radiological courses of symptomatic spontaneous isolated superior mesenteric artery dissection (SISMAD) after conservation, clarify the relationship between its morphological change and initial imaging classification, and identify these factors that affect dissection remodeling.

**Methods:**

Eighty-nine conservative patients with SISMAD who underwent periodic follow-up of computed tomography angiography (CTA) were enrolled. Initial morphologic classification, imaging features and dissection remodeling were analyzed retrospectively. Logistic regression was used to identify predictors for remodeling. Receiver operating characteristics were performed for cutoff threshold.

**Results:**

Zerbib classification was adapted and initial CT appearance divided eighty-nine patients into: type I (15.7%), patent false lumen (FL) with both entry and re-entry; type II (37.1%), “cul-de-sac” shaped FL without re-entry; type III (27.0%), thrombosed FL with ulcer-like-projection (ULP); type IV (18%), intramural hematoma; type V (0%), dissecting aneurysm; and type VI (2.2%), total or partial occlusion of superior mesenteric artery (SMA). Follow-up CTA revealed complete remodeling (33.7%), partial remodeling (16.9%), no change (25.8%), type change (13.5%) and dissection progression (10.1%). There was no dissection-related mortality. Type I (92.9%) sustained patent FL and no angiographic change. Type II showed partial remodeling (42.4%), no change (27.3%) and dissection progression (27.3%), and the length of FL enhancement positively predicted dissection progression with the cutoff of 40.3 millimeters. Type III achieved complete remodeling (58.3%) or evolved into type II (41.7%), and the distance between SMA orifice and ULP negatively predicted type change with the cutoff of 23.5 millimeters. Type IV (87.5%) achieved complete remodeling due to hematoma absorption. One patient underwent stent placement for the evolution of ULP into an enlarged blind-ending FL 2 months after conservation.

**Conclusion:**

After conservation, patent FL with a distal re-entry is no morphological change, FL thrombosis tends to be resolved, and the “cul-de-sac” shaped FL without re-entry is partially shortened, no change or progressively dilated. FL enhancement length ≥ 40.3 millimeters is a predictor for the blinding-end FL enlargement. Thrombosed FL with ULP evolves into a patent “cul-de-sac” shaped FL when the distance between SMA orifice to ULP is less than 23.5 millimeters. A careful follow-up is necessary for the lesions with demonstrated predictors.

## Introduction

Spontaneous isolated superior mesenteric artery dissection (SISMAD) is a rare vascular disorder but potentially fatal pathology (1, 2). A classic autopsy series reported that the incidence of superior mesenteric artery (SMA) dissection was approximately 0.08% (3). However, there has been a dramatic increase in reports of the disease, especially from East Asian Country since improvements in the technology and the widespread use of computed tomography angiography (CTA) for acute abdominal pain (4).

The treatment options for patients with SISMAD range from conservative therapy to endovascular intervention or open surgical treatment. Open surgery is only necessary when intestinal necrosis or aneurismal rupture bleeding (5, 6). Endovascular stenting has been proposed as a treatment option for patients with severe true lumen (TL) stenosis (>75%), large dissecting aneurysm and persistent abdominal pain despite maximum conservative management (7). Most patients showed clinical improvement after conservative treatment with or without anticoagulation or anti-platelet therapy (8–12). Many studies have suggested that conservative treatment should be considered as a first line treatment for SISMAD patients without evidence of bowel ischemia or infarction on initial CT scan (8–12).

CTA is the imaging modality of choice in the diagnosis and treatment strategies of SISMAD and can clearly demonstrate the location and extent of dissection. The systematic analysis of CT scans reveals detailed imaging features of SISMAD consisting of entry and re-entry location, dissection length, TL stenosis, false lumen (FL) patency, and ulcer like-projection (ULP). Patients with SISMAD may have different angiographic types of dissections, progress with different results, and require different management. The classification of SISMAD types appears to be important for treatment selection and prognosis prediction.

The morphologic course of SISMAD and its relationship with initial CT classification after conservative management have not yet been firmly established. Predictive factors for worsening dissection progression or unusual type change are not fully understood. Therefore, the purpose of the study is to examine the serial radiologic changes of patients with SISMAD who underwent conservative management, to define a correlation between morphologic classification of SISMAD on CTA and natural course, and to identify CT angiographic features predictive of dissection progression and type change.

## Materials and Methods

### Study Population

A retrospective observational study was conducted on patients with symptomatic SISMAD at a single institution over the past 6 years from January 2015 to March 2022. Eighty-nine patients who underwent conservative treatment and periodic follow-up CTA were included in the study. The diagnosis of SISMAD was confirmed when one of the following signs was seen on the initial CT: (1) intimal flap and contrast enhancement within the FL; (2) crescent-shaped area along the wall of SMA with higher attenuation than blood, showing no contrast enhancement after contrast material injection. The dissection was not related to trauma, abdominal surgery, or other interventions on the artery. Patients with abdominal aortic dissection, SMA thrombosis or aneurysmal formation without dissection were excluded. This study was approved by the institutional review boards of Wuhan Union Hospital according to the ethical guidelines of the 1975 Declaration of Helsinki and its amendments. Because this was a retrospective review, written informed consent was waived and patient’s information were anonymized before analysis.

### Analysis and Measurement of SISMAD by CT

We retrospectively reviewed the clinical features, risk factors, CT images, treatment modalities and follow-up outcomes of 89 patients. Imaging characteristics such as morphologic classification, location of entry and re-entry site, origin of dissection, patency of the FL, ULP and aneurismal formation were analyzed on initial and follow-up CTA. Dissection length, TL minimum diameter, FL maximum diameter, length of the FL enhancement, distance between the SMA orifice and dissection origin, distance from the maximum curve of the SMA to dissection origin, SMA branching angle from aorta, ULP length, ULP depth, distance between the SMA orifice and ULP, and distance from the maximum curve of SMA to ULP were measured in this study. The percent compression of the TL was determined by CT images based on the luminal diameter of adjacent normal SMA and the diameter of the TL with maximum stenosis between the orifice of SMA and the origin of ileocolic artery.

### Classification of SISMAD by CT

SISMAD was classified into six types according to the configuration on CT scans. Four vascular surgeons reviewed the axial and reconstructed sagittal views of contrast-enhanced CT scans and classified the SISMAD separately. If there were any discrepancies, all four members discussed the cases and reached agreement. The morphology after SISMAD was classified into the following six types based on the classification described by Sakamoro et al. (13) and modified by Zerbib et al. (14): Type I, patent FL with both entry and re-entry; Type II, “cul-de-sac” shaped FL without re-entry; Type III, thrombosed FL with ULP; Type IV, completely thrombosed FL without ULP; Type V, dissecting aneurysm; Type VI, SMA dissection with total occlusion (VIa) or partial thrombosis (VIb) of SMA. Dissecting aneurysm formation was defined as arterial dilation at least 1.5 times larger than normal mesenteric artery adjacent to the SISMAD.

### Treatment Strategies

Conservative management comprised blood pressure control (target systolic and diastolic blood pressures of <140 and <90 mmHg, respectively), bowel rest, parenteral nutritional support, and pain management. The duration of bowel rest was dependent on symptom resolution, and bowel rest was usually discontinued one or 2 days after complete symptom resolution. All patients received parenteral nutritional support until bowel rest. The use of antithrombotic therapy was based on doctor preference and was not compulsory for all patients. A close surveillance range of five to 7 days was used. After close surveillance, follow-up CTA was repeated to evaluate the morphological changes of the dissection, including dissection length, dissecting aneurysm formation, and TL stenosis. Conservative treatment was continued for patients who experienced symptom relief and were without morphological worsening of the dissection. For patients who experienced persistent (symptom duration of greater than 7 days) or aggravated symptoms or patients with follow up CTA findings suggestive of worsening dissection (including newly detected dissecting aneurysms and severe TL stenosis), subsequent angiography was performed to confirm deterioration, and then endovascular stenting was performed. Severe TL stenosis was defined as luminal narrowing of >75% of the normal mesenteric artery diameter.

### Follow-Up and Result Classification

Symptomatic patients who underwent conservative management were periodically followed up using CT angiography for up to 6 years after diagnosis. The frequency of performing CT depended on each patient, although it was normally performed every 1–3 months in the first half of the year and every 6 months thereafter. Serial morphologic changes in the SMA lesion focused on the extent of distal or proximal progression of the arterial dissection, occlusion or remodeling of the FL and TL, or aneurysmal changes of the FL. The CT results on follow-up were compared with the initial findings and were classified according to the following (2, 8, 9): (1) Complete remodeling was defined as the absence of residual arterial dissection and the absence of arterial narrowing on the follow-up CT image (2, 8, 9). (2) Partial remodeling was defined as improved luminal patency of the SMA but with luminal narrowing or arterial dissection on the follow-up CT image (2, 8, 9). (3) Aneurysmal dilation was defined as focal SMA dilation >150% of the normal SMA adjacent to the dissection (9). (4) Morphologic progression was defined as the aggravation of stenosis of the TL or aneurysmal degeneration of the FL (2). (5) Type change was defined as change in configuration from one type to another (8).

### Statistical Analysis

All statistical analyses were performed using SPSS version software (SPSS Inc., Chicago, IL, United States). Categorical variables were presented as number and the percentage of patients and compared by Fisher’s exact test. Continuous variables were expressed as mean ± standard deviation with normal distribution or median and interquartile range in case of skewness distribution. Univariate analysis between group was performed by the student *t*-test for variables with normal distribution and the Mann–Whitney *U* test for independent non-parametric data. Differences among groups were analyzed by ANOVA for variables with normal distribution and Kruskal–Wallis test for independent non-parametric data.

Multivariate ordinal logistic regression was performed to assess the independent association between predictors and morphologic progression in type II dissection of “cul-de-sac” shaped FL without re-entry. Binary logistic regression was used to evaluate the different variables and their relation to type change in type IV dissection of completely thrombosed FL without ULP. Receiver operating characteristic (ROC) analyses were performed to evaluate the prediction potential of each of the CT parameters related to dissection remodeling. Areas under the curve (AUCs) were determined and cut-off values were chosen that maximized the Youden-criterion for each parameter. Sensitivities, specificities, and prediction accuracies were determined with regard to each cutoff. A *p* value <0.05 indicated a statistically significant difference.

## Results

### Clinical Characteristics

Between January 2015 to March 2022, 89 patients of SISMAD who underwent conservative management and periodic follow-up of CTA were admitted. Patient demographics, clinical presentations, comorbidities, and classification of the enrolled patients were summarized in Table 1. 74 (83.1%) patients were male with a median age of 51.7 years ranging from 47 to 55 years. 78 (87.6%) patients had symptoms of abdominal pain. Other concomitant symptoms included nausea and vomiting in 17 (19.1%) patients and blood stools in 7 patients (7.9%). Coexisting medical conditions included hypertension in 32 (36.0%) patients, smoking in 32 (36.0%) patients, hyperlipidemia in 22 (24.7%) patients, and diabetes in 3 (3.4%) patients.

**TABLE 1:** Demographic data and clinical features of patients with SISMAD.

	Patients (*n* = 89)
Age, years	51.7 (47; 55)
Male, *n* (%)	74 (83.1%)
**Clinical manifestation, *n* (%)**	
Abdominal pain	78 (87.6%)
Nausea/Vomiting	17 (19.1%)
Bloody stools	7 (7.9%)
**Co-morbidities, *n* (%)**	
Hypertension	32 (36.0%)
Tobacco Use (Current or ex-smoker)	32 (36.0%)
Hyperlipidemia	22 (24.7%)
Diabetes mellitus	3 (3.4%)
Chronic kidney disease	0 (0%)
Coronary artery disease	1 (1.1%)
History of abdominal surgery	10 (11.2%)
**Dissection characteristics**	
Distance between the SMA orifice and dissection origin, mm	12.0 (7.8; 25.5)
The length of dissection, mm	69.2 ± 33.3
True lumen residual diameter, mm	3.3 ± 1.9
Adjacent normal SMA size, mm	7.1 ± 1.2
Percent of true lumen stenosis	42.1% (28.5%; 59.5%)
SMA branching angle from aorta	113.2 ± 22.8

*Continuous values are presented as mean ± standard deviation or median (first quartile; third quartile), according to the normality in distribution. Categorical data are shown as number (% of each group). SISMAD, spontaneous isolated superior mesenteric artery dissection; SMA, superior mesenteric artery.*

As measured on initial CT angiographic images, the distance between SMA origin and dissection origin was 12.0 (7.8; 25.5) mm, the length of dissection was 69.2 ± 33.3 mm, TL residual diameter was 3.3 ± 1.9 mm, adjacent normal SMA size was 7.1 ± 1.2 mm, the percentage of the maximum TL stenosis was 42.1% (28.5%; 59.5%), and SMA branching angle from aorta was 113.2 ± 22.8.

### In-Hospital and Follow-Up Clinical Outcomes

**FIGURE 1:**
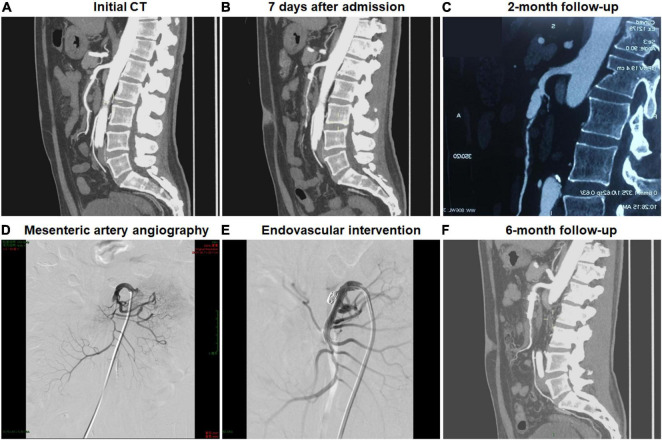
Endovascular coiling assisted stent placement and follow-up computed tomography (CT) for type III developed into type II spontaneous isolated superior mesenteric artery dissection. **(A,B)** Initial and 1-week CT angiography showed the thrombosed false lumen with ulcer-like projection in a 52-year-old man. **(C)** Two months later, type III lesion evolved into type II dissection with a patent “cul-de-sac” false lumen but no re-entry, and the true lumen was severely compressed. **(D)** Mesenteric artery angiography revealed the dissection formation and the occlusion of the main trunk. **(E)** Type II dissection was treated using stent placement and coil embolization *via* stent mesh. **(F)** Six month later, the stent remained patent.

In all patients, clinical symptoms, including abdominal pain, resolved or markedly improved within the hospitalization. The duration of clinical follow-up ranged from 1.2 to 55.3 months, and the mean duration of follow-up was 8.0 months. There was not dissection-related mortality during follow-up, and no open surgery was needed. One patient was a 52-year-old man and presented with sudden-onset abdominal pain and bloody stools 2 days before admission. Initial CTA indicated the thrombosed FL with ULP diagnosed as type IV lesion (Figure 1). Two months after conservative management, this patient was re-admitted because of recurrent abdominal pain, displayed type II lesion of a patent “cul-de-sac” shaped FL without re-entry on follow-up CT scans and underwent endovascular coil assisting stent treatment (Figure 1). In the initially conservative patient, ULP progressively dilated and evolved into a patent FL without re-entry during the follow-up, and a blind-ending FL might rapidly enlarge, thus causing severe stenosis of TL and bowel ischemia.

### In-Hospital Classification and Follow-Up Radiographic Findings

Classification on initial CT and follow-up morphologic change of SISMAD were summarized on Table 2. SISMAD was categorized into the following types according to Zerbib’s classification: 14 (15.7%) type I, 33 (37.1%) type II, 24 (27.0%) type III, 16 (18.0%) type IV, 0 (0%) type V, and 2 (2.2%) type VI.

**TABLE 2:** Follow-up computed tomography results of conservative treatment according to the angiographic findings.

	All patients	Type I	Type II	Type III	Type IV	Type VI	*P*-value
Conservative management	89	14	33	24	16	2	
Complete remodeling	30 (33.7%)	1 (7.1%)	1 (3.0%)	14 (58.3%)	14 (87.5%)	0 (0%)	<0.001
Partial remodeling	15 (16.9%)	0 (0%)	14 (42.4%)	0 (0%)	0 (0%)	1 (50%)	<0.001
No change	23 (25.8%)	13 (92.9%)	9 (27.3%)	0 (0%)	0 (0%)	1 (50%)	<0.001
Type change	12 (13.5%)	0 (0%)	0 (0%)	10 (41.7%)	2 (12.5%)	0 (0%)	<0.001
Morphologic progression	9 (10.1%)	0 (0%)	9 (27.3%)	0 (0%)	0 (0%)	0 (0%)	0.003

*Categorical data are shown as number (% of each group).*

Morphologic changes of SISMAD were classified into the following types based on following CTA scans: 30 (33.7%) complete remodeling, 15 (16.9%) partial remodeling, 23 (25.8%) no change, 12 (13.5%) type change, and 9 (10.1%) dissection progression. No dissection aneurysmal change (1.5 times larger than the normal adjacent mesenteric artery) or SMA rupture was detected during the follow-up period.

**FIGURE 2:**
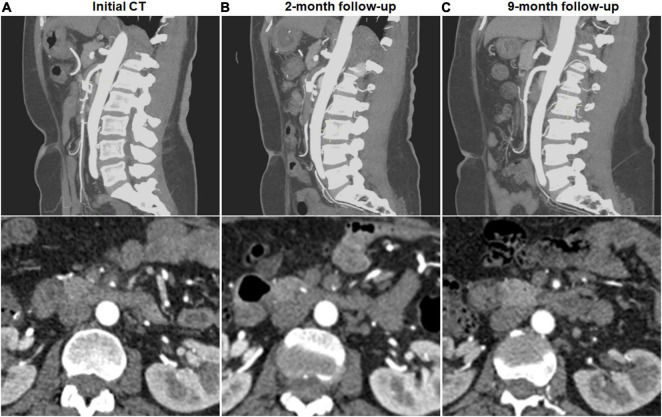
Complete remodeling of type III spontaneous isolated superior mesenteric artery dissection on follow-up computed tomography (CT) angiography. **(A)** Initial CT angiography showed the thrombosed false lumen with ulcer-like projection (ULP) in a 52-year-old woman. **(B)** Two months later, stenosis of true lumen was aggravated with thrombosis of false lumen and ULP. **(C)** Nine months later, ULP disappeared and thrombosis in the false lumen was completely resolved.

**FIGURE 3:**
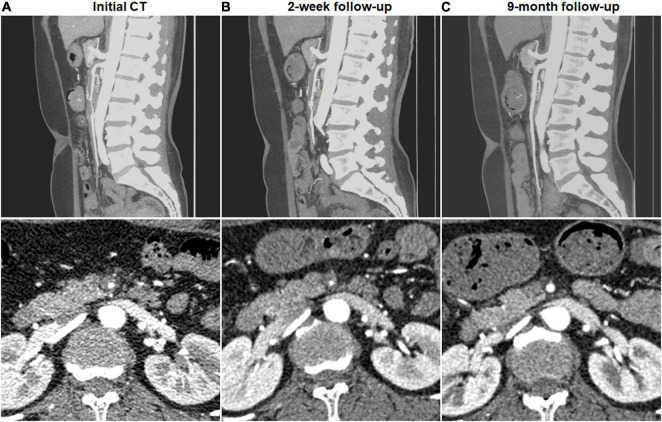
Complete remodeling of type IV spontaneous isolated superior mesenteric artery dissection on follow-up computed tomography (CT) angiography. **(A)** Initial CT angiography showed the completely thrombosed false lumen in a 44-year-old woman. **(B)** Two weeks later, false lumen thrombus became thickened, and stenosis of true lumen was aggravated. **(C)** Nine months later, false lumen thrombus was completely resolved, and type IV dissection achieved complete remodeling.

Complete remodeling on CTA occurred in 1 (7.1%) of 14 type I dissections of patent FL with both entry and re-entry, 1 (3.0%) of 33 type II dissections of patent “cul-de-sac” shaped FL without re-entry, 14 (58.3%) of 24 type III dissections of thrombosed FL with ULP (Figure 2) and 14 (87.5%) of 16 type IV dissections of the completely thrombosed FL without ULP (Figure 3). In 14 type III lesions, the mean diameter of TL increased from 3.2 (3.0; 4.1) mm on initial CT to 5.8 ± 1.0 mm on follow-up CT (*p* < 0.001). In 14 type IV lesions, the mean diameter of TL was recovered from 4.3 ± 1.1 mm on initial CT to 6.6 ± 1.0 mm on follow-up CT (*p* < 0.001). Thus, thrombus in the FL of type III and IV lesions may contribute to the process of the complete wall remodeling.

**FIGURE 4:**
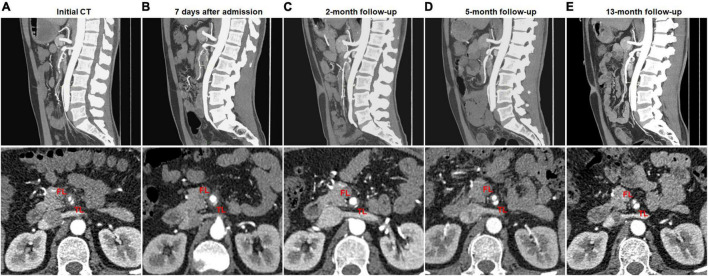
Partial remodeling of type II spontaneous isolated superior mesenteric artery dissection on follow-up computed tomography (CT) angiography. **(A)** Initial CT angiography showed the patent “cul-de-sac” shaped false lumen without re-entry in a 43-year-old man. **(B,C)** One week and 2 months after conservative treatment, the false lumen was dilated and severely compress the true lumen. **(D,E)** Five and 13 months later, the false lumen was progressively reduced, and the true lumen was partially recanalized.

Partial remodeling on CTA was noted in 14 (42.4%) of 33 type II dissections of patent “cul-de-sac” shaped FL without re-entry (Figure 4) and 1 (50%) of 2 type V dissections with occlusion of SMA main trunk. At 14 type II lesions, the mean diameter of TL increased from 2.6 ± 1.0 mm on initial CT to 4.9 ± 0.7 mm on follow-up CT (*p* < 0.001), and the mean diameter of FL was reduced from 6.1 ± 1.5 mm on initial CT to 4.9 ± 1.6 mm on follow-up CT (*p* = 0.020).

**FIGURE 5:**
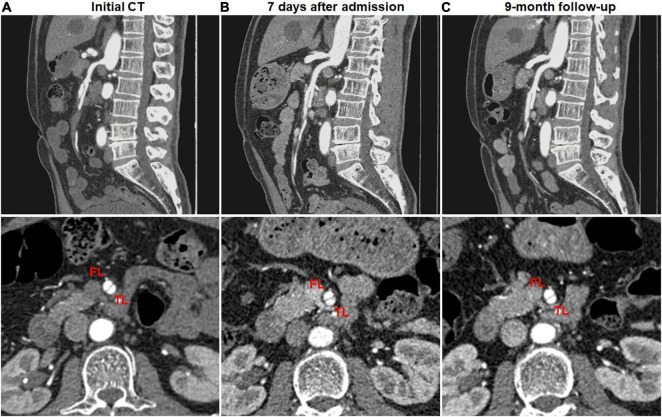
No change of type I spontaneous isolated superior mesenteric artery dissection on follow-up computed tomography (CT) angiography. **(A)** Initial CT angiography showed patent false lumen and true lumen with both entry and re-entry in a 53-year-old man. **(B)** One week after conservative management, type I dissection showed no interval change. **(C)** Nine month later, patent false lumen was sustained with both entry and re-entry as well as no aneurysmal dilatation.

**FIGURE 6:**
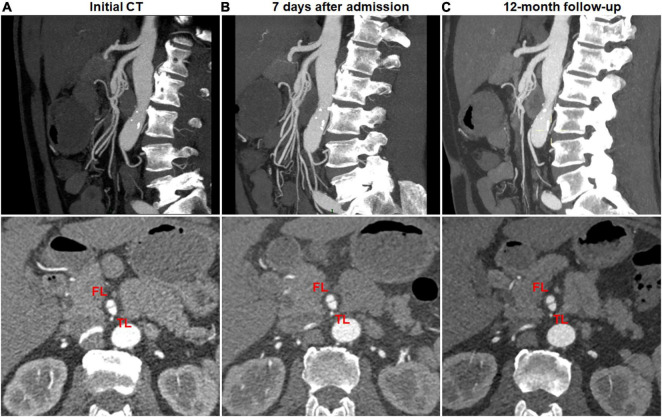
No change of type II spontaneous isolated superior mesenteric artery dissection on follow-up computed tomography (CT) angiography. **(A)** Initial CT angiography showed the patent “cul-de-sac” shaped false lumen without re-entry in a 66-year-old woman. **(B)** One week after conservative management, true and false lumens were relatively stable. **(C)** The true lumen and blind-ending false lumen remained unchanged during the follow-up period of 12 months.

No change on CTA was observed in 13 (92.9%) of 14 type I dissections of patent FL with both entry and re-entry (Figure 5), 9 (27.3%) of 33 type II dissections of “cul-de-sac” shaped FL without re-entry (Figure 6), and 1 (50%) of 2 type V dissections with occlusion of SMA main trunk. In 13 type I lesions, there was no obvious difference on the mean diameter of TL and FL between initial and follow-up CT (3.3 ± 1.5 *vs.* 3.3 ± 1.2 mm for TL, *p* = 0.962; 6.0 ± 1.6 *vs.* 6.1 ± 1.7 mm for FL, *p* = 0.939). In 9 type II lesions, no difference was observed on the mean diameter of TL and FL between initial and follow-up CT (3.8 ± 1.1 *vs.* 3.9 ± 1.2 mm for TL, *p* = 0.930; 7.7 ± 1.3 *vs.* 7.6 ± 1.2 mm for FL, *p* = 0.978). Type I lesions showed a tendency for no change on follow-up angiograms.

**FIGURE 7:**
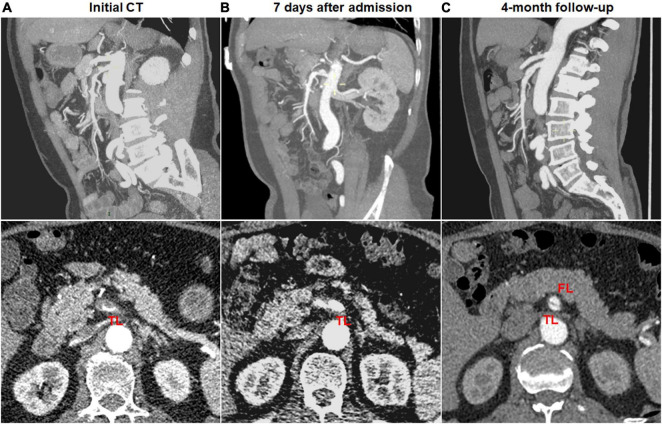
Type change of type III spontaneous isolated superior mesenteric artery dissection on follow-up computed tomography (CT) angiography. **(A)** Initial CT angiography showed the thrombosed false lumen with ulcer-like projection (ULP) in a 49-year-old man. **(B)** One week after conservative management, the thrombosis of false lumen was partially resolved and true lumen was slightly dilated. **(C)** Follow-up angiogram at 4 months after conservative treatment showed double-lumen sign on an axial view and a patent “cul-de-sac” shaped false lumen without re-entry on a sagittal view (type II dissection).

Type change on CTA was noted in 10 (41.7%) of 24 type III dissections of thrombosed FL with ULP and 2 (12.5%) of 16 type IV dissections of the completely thrombosed FL. 10 type III lesions evolved into type II dissections of “cul-de-sac” shaped FL without re-entry (Figure 7), and the stenosis of TL was improved with hematoma regression. The mean diameter of TL increased from 3.6 ± 1.2 mm on initial CT to 5.9 ± 1.3 mm on follow-up CT (*p* = 0.001). 1 type III lesion developed into type II with TL severely compressed and received stent assisted coiling (Figure 1).

**FIGURE 8:**
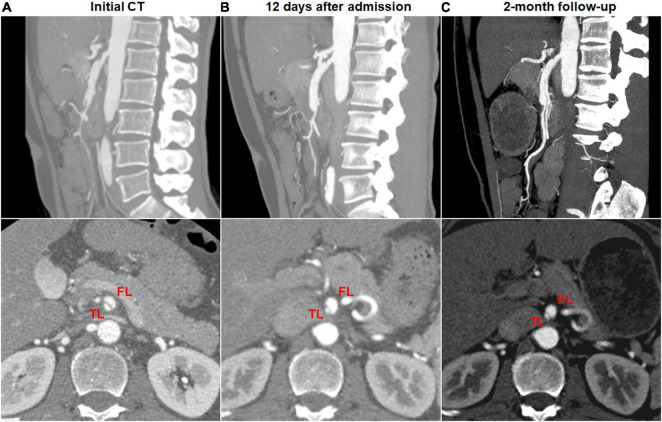
Morphologic progression of type II spontaneous isolated superior mesenteric artery dissection on follow-up computed tomography (CT) angiography. **(A)** Initial CT angiography showed the patent “cul-de-sac” shaped false lumen without re-entry in a 48-year-old man. **(B)** Twelve days later, blind-ending false lumen was prolonged and compressed the true lumen despite symptomatic relief. **(C)** Two months later, the false lumen was further enlarged, and true lumen remained severely stenosed.

Dissection progression on CTA occurred in 9 (27.3%) of 33 type II dissections of “cul-de-sac” shaped FL without re-entry (Figure 8). The mean diameter of TL declined from 2.8 ± 0.7 mm on initial CT to 1.3 (1.1; 1.7) mm on follow-up CT (*p* = 0.003). The mean diameter of FL increased from 5.3 ± 0.7 mm on initial CT to 7.2 ± 1.0 mm on follow-up CT (*p* < 0.001).

### Predictive Factors for Dissection Progression and Type Change

Candidate predictive factors for morphologic progression on type II dissections were summarized in Table 3. Type II lesions achieved partial remodeling, no change, and morphologic progression in the percentage of 42.4%, 27.3%, and 27.3%, respectively. Multivariate ordinal logistic regression analysis was performed to test the association between dissection progression and predictive factors. The length of FL enhancement was longer in dissection progression (46.1 ± 16.1 mm) compared to no change (31.2 ± 8.0) and partial remodeling [15.4 (13.7; 20.1)] (*p* < 0.001), and was an independent predictor factor for morphologic progression in type II lesions (*p* = 0.001; 95%CI, 0.070–0.268). The distance between SMA orifice and dissection origin did not statistically differ in dissection progression (29.5 ± 18.4 mm), no change (34.9 ± 14.4) and partial remodeling (24.3 ± 13.5) (*p* = 0.276). Similarly, there were no significant difference on the distance from the maximum curve of the SMA to the dissection among dissection progression (16.8 ± 7.3 mm), no change (19.6 ± 9.4) and partial remodeling (11.8 ± 8.0) (*p* = 0.091). The distance between SMA orifice and dissection origin (*p* = 0.218; 95%CI, −0.038–0.164) and the distance from the maximum curve of the SMA to the dissection (*p* = 0.666; 95%CI, −0.201–0.129) were not independent predictors of morphologic progression in type II lesions. The percentage of TL stenosis was less in dissection progression (24.5% ± 5.8%) compared to no change (28.9% ± 6.9%) and partial remodeling (37.0% ± 14.9%) (*p* = 0.034), but was not found to be an independent predictor of morphologic progression in type II lesions (*p* = 0.087; 95%CI, −22.593–1.538).

**TABLE 3:** Determinant affecting morphological progression in Type II SISMAD with “cul-de-sac” shaped FL without re-entry.

	Partial remodeling	No change	Morphologic progression	Univariate analysis	Multivariate ordinal logistic regression	
				*P*-value	95% CI	*P*-value
Length of the FL enhancement, mm	15.4 (13.7; 20.1)	31.2 ± 8.0	46.1 ± 16.1	<0.001	0.070–0.268	0.001
Distance between the SMA orifice and dissection origin, mm	24.3 ± 13.5	34.9 ± 14.4	29.5 ± 18.4	0.276	−0.038–0.164	0.218
Distance from the maximum curve of the SMA to the dissection, mm	11.8 ± 8.0	19.6 ± 9.4	16.8 ± 7.3	0.091	−0.201–0.129	0.666
Percent of the TL stenosis (%)	37.0 ± 14.9	28.9 ± 6.9	24.5 ± 5.8	0.034	−22.593–1.538	0.087

*Continuous values are presented as mean ± standard deviation or median (first quartile; third quartile), according to the normality in distribution. SISMAD, spontaneous isolated superior mesenteric artery dissection; FL, false lumen; SMA, superior mesenteric artery; CI, confidence interval; TL, true lumen.*

Potential predictive factors for type change on type III dissections were summarized in Table 4. Type III lesions achieved complete remodeling and type change in the percentage of 58.3% and 41.7%, respectively. Binary logistic regression analysis was performed to test the association between type change and potential predictive factors. The distance between SMA orifice and ULP was statistically less in type change [10.3 (5.1; 25.7) mm] compared to complete remodeling [32.6 (27.5; 38.8)] (*p* = 0.016), and was an independent negative predictor of change in type III lesions (*p* = 0.046; OR, 0.842; 95%CI, 0.711–0.997). No significance difference was seen in the length and depth of ULP between type change and complete remodeling [9.4 (7.0; 12.3) *vs.* 11.3 ± 3.9 mm for the length of ULP, *p* = 0.437; 5.0 (3.6; 5.3) *vs.* 4.8 ± 1.3 mm for the depth of ULP, *p* = 0.709]. The length (*p* = 0.513; OR, 1.133; 95%CI, 0.779–1.647) and depth (*p* = 0.160; OR, 0.218; 95%CI, 0.026–1.826) of ULP were found not to be the significant predictor factors of type change. Distance from the maximum curve of the SMA to the ULP was statistically shorter in type change [6.5 (5.6; 9.8)] compared to complete remodeling [10.2 (8.2; 14.6)] (*p* = 0.016), but was not found to be an independent predictor of change in type III lesions (*p* = 0.924; OR, 0.984; 95%CI, 0.709–1.366).

**TABLE 4:** Determinant inducing type change in Type III SISMAD with thrombosed FL with ULP.

	Complete remodeling	Type change	Univariate analysis	Binary logistic regression		
			*P*-value	OR	95% CI	*P*-value
Distance between the SMA orifice and ULP, mm	32.6 (27.5; 38.8)	10.3 (5.1; 25.7)	0.016	0.842	0.711–0.997	0.046
Length of the ULP, mm	11.3 ± 3.9	9.4 (7.0; 12.3)	0.437	1.133	0.779–1.647	0.513
Depth of the ULP, mm	4.8 ± 1.3	5.0 (3.6; 5.3)	0.709	0.218	0.026–1.826	0.160
Distance from the maximum curve of the SMA to the ULP, mm	10.2 (8.2; 14.6)	6.2 (5.6; 9.8)	0.016	0.984	0.709–1.366	0.924

*Continuous values are presented as mean ± standard deviation or median (first quartile; third quartile), according to the normality in distribution. SISMAD, spontaneous isolated superior mesenteric artery dissection; FL, false lumen; ULP, ulcer like-projection; SMA, superior mesenteric artery; OR, odd ratio; CI, confidence interval.*

### ROC Analysis of Predictor Factors for Dissection Remodeling

**FIGURE 9:**
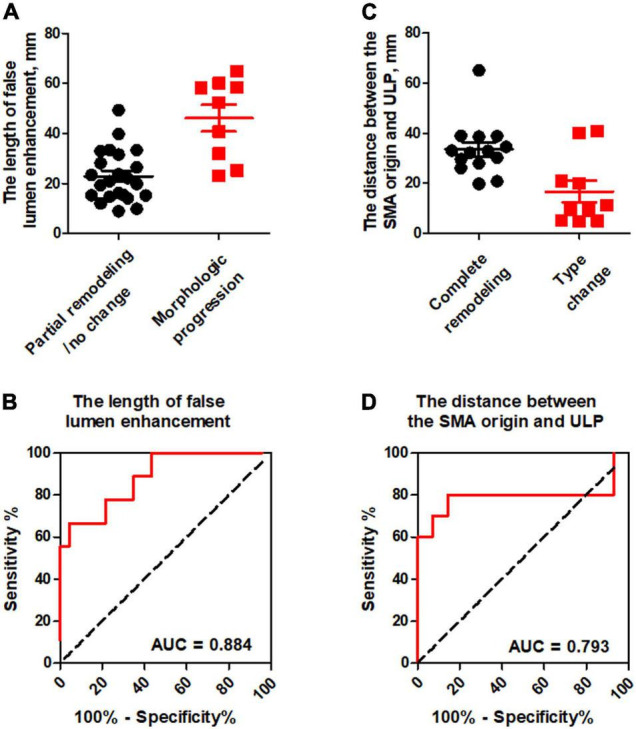
ROC analysis for the prediction of dissection remodeling by the initial CT parameters. **(A)** The length of false lumen enhancement was statistically higher in morphologic progression than in partial remodeling/no change. **(B)** ROC curves indicated that the length of FL enhancement was an independent predictor for dissection progression in type II dissection with a patent “cul-de-sac” false lumen but no re-entry with the AUC of 0.884. **(C)** The distance between the SMA orifice and ULP was significantly lower in type change than in complete remodeling. **(D)** ROC curves revealed that the distance between the SMA orifice and ULP had the AUC of 0.793 and negatively predicted the type change in type III lesion with thrombosed false lumen with ULP.

ROC analysis of the ability of the initial CT parameters to predict dissection remodeling in type II and III lesions was shown in Table 5. For type II lesions, the length of FL enhancement was significantly higher in morphologic progression than in partial remodeling/no change (*p* < 0.001) (Figure 9A). AUC with the length of FL enhancement was 0.884 (Figure 9B). The optimal threshold of the length of FL enhancement for predicting morphologic progression on type II dissections, as determined by ROC analysis, was 40.3 millimeters (Table 3). The sensitivity, specificity, and accuracy of the length of FL enhancement for predicting morphologic progression was 66.7%, 95.7%, and 87.9% (Table 3).

**TABLE 5:** ROC analysis of the predictor CT parameters determining the dissection progression in Type II SISMAD with “cul-de-sac” shaped FL without re-entry and type change in Type III SISMAD with thrombosed FL with ULP.

Predictor parameters	AUC	SE of area	95% CI	*P*-value	Cut-off	Sensitivity (%)	Specificity (%)	Accuracy (%)
Length of the false lumen enhancement, mm	0.884	0.064	0.758–1.010	<0.001	40.3	66.7%	95.7%	87.9%
Distance between the SMA orifice and ULP, mm	0.793	0.118	0.562–1.024	0.016	23.5	85.7%	80.0%	83.3%

*ROC, receiver operating characteristics; CT, computed tomography; SISMAD, spontaneous isolated superior mesenteric artery dissection; FL, false lumen; ULP, ulcer like-projection; SMA, superior mesenteric artery; AUC, area under the curve; SE, standard error; CI, confidence interval.*

For type III lesions, the distance between the SMA orifice and ULP was significantly lower in type change than in complete remodeling (*p* = 0.016) (Figure 9C). The distance between SMA origin and ULP had the best test performance to predict type change on type III dissections under the AUC of 0.793 (Figure 9D). A distance of 23.5 millimeters or less was the best cut-off to predict type change, which resulted in a sensitivity of 85.7%, a specificity of 80.0%, and an accuracy of 83.3% (Table 3).

## Discussion

SISMAD is rare cause of acute abdominal pain, and occurs mainly in male patients in their fifth decade. In this study, 83.1% of patients with SISMAD were male, and mean age of the patients was 51.7 years. Overall, 89.6% of the patients presented with sudden-onset severe abdominal or back pain. Regarding of the causes of SISMAD, hemodynamic abnormalities in the SMA at the transitional point from the fixed to relatively mobile segment of SMA at the lower margin of the pancreas is a major cause of SMA dissection (15), which is analogous to the aortic dissection that starts just distal to the left subclavian artery. The location of the entry point of dissection is usually located at 1.5–3 cm distal to the orifice of SMA (16). Unusual mechanical stresses on the anterior wall of this portion may be an important factor in the development of SISMAD. Smoking have been indicated as possible etiologies, which is consistent with the current case. In our series, approximately 36.0% of the present patients had the history of smoking. In contrast to the well-known correlation between hypertension and cortic dissection, hypertension was only present in 36% of our patient group. Diabetes mellitus, as a risk factor for atherosclerosis, were identified in 3.4% of patients, which was not at least potential etiological factors in the development of SISMAD. Of 622 Chinese patients with SISMAD reported in Chinese-language literature, diabetes (6%) was obviously less prevalent than hypertension (42.7%) and smoker (19.6%) (2).

Various classifications based on imaging characteristics have been proposed to guide treatment and to evaluate prognosis. Sakamoto et al. (13) categorized SISMAD into four types based on the patency of the FL: type I, patent FL with both entry and re-entry; type II, “cul-de-sac”-shaped FL without re-entry; type III, thrombosed FL with an ulcer-like projection (ULP); and type IV, completely thrombosed FL without ULP. However, this classification does not take into consideration the patency of the TL, which is valuable for the evaluation of the mesenteric blood supply. Based on Sakamoto’s classification, Yun et al. (17) reported another categorization as follows: type I, patent TLs and FLs with visible entry and re-entry sites; type IIa, patent TLs and FLs with visible entry but no visible re-entry (blind pouch of FL); type IIb, patent TL but thrombosed FL without visible re-entry; type III, SMA dissection with occluded TLs and FLs. This classification added the type “total occlusion of SMA,” but did not include “ULP” that is often seen in SISMAD patients. Therefore, neither Sakamoto nor Yun classifications have established a clear relationship between radiological appearance and clinical course (13, 17). Zerbib et al. introduced a modified classification including the description of “dissecting aneurysm,” “thrombosis of SMA” and “ULP” (14). Our article adopted the Zerbib classification, which was found to be more relevant to the SISMAD clinical features. In the present study, the CT finding classified patients into type I (15.7%, 14/89), type II (37.1%, 33/89), type III (27.0%, 24/89), type IV (18.0%, 16/89), type V (0%,0/67), and type VI (2.2%, 2/89).

The natural course of SISMAD has not been well known. We reported our observation regarding correlation between morphologic types of SISMAD on CTA and natural course. We founded complete remodeling (33.7%, 30/89), partial remodeling (16.9%, 15/89), no change (25.8%, 23/89), type change (13.5%, 12/89), and dissection progression (10.1%, 9/89). Complete remodeling was more likely to occur in patients with thrombosed FL with ULP (58.3%, 14/24) and intramural hematoma (87.5%, 14/16). Partial remodeling tended to occur in patients with a shorter patent “cul-de-sac” FL but no re-entry (42.4%, 14/33). No change was observed in patients with patent entry and re-entry (92.9%, 13/14) and patent “cul-de-sac” FL of intermediate length but no re-entry (27.3%, 9/33). Type change was explained as the fact that type II with patent “cul-de-sac” FL but no re-entry originated from type III of thrombosed FL with ULP (41.7%, 10/24) and type IV of completely thrombosed FL (12.5%, 2/16). Dissection progressed in patients with a longer patent “cul-de-sac” FL but no re-entry (27.3%, 9/33).

There were 13 (92.9%) patients of 14 Zerbib type I SISMAD, in which serial CTA found sustained patent FL and no angiographic changes after successful conservative treatment. No change on follow-up CTA is similar to a published report by Kim et al. (18) on the natural history of SISMAD with both entry and re-entry. In their study, CTA follow-up in 5 patients with a patent FL did not demonstrate any definite changes compared with the initial CT scan with a median time of 17.1 months (18). Park et al. reported that SISMAD with a patent FL showed a propensity for no change on follow-up CTA (10), consistent with our results. In cases with a distal re-entry, blood flow pressure can be released through the patent TLs and FLs of SMA.

Zerbib type II SISMAD was the most frequent type in this study (37.1%, 33/89) and showed partial remodeling (42.4%, 14/33), no change (27.3%, 9/33), and dissection progression (27.3%, 9/33) during follow-up. The length of FL enhancement in type II dissection is observed to be the main determinant affecting morphological alteration after conservative management. When the length of FL enhancement was more than 40.3 millimeters, Type II patients of blinding-ending FL had a high risk of the enlargement of the FL and the compression of the TL, and should be intensively monitored on abdominal symptom relief during conservative management. According to Poiseuille’s law of fluid dynamics, blood flow pressure is positively proportional to the length of configuration. Thus, persistent blood flow filling through entry tear maintains the continued pressurization in the FL without a distal re-entry, and may lead to its expansion. Previous study demonstrated that a dissection length ≥ 50 mm was an independent risk factor for failed conservative treatment (19). The dissection length is also positively correlated with pain severity in SISMAD patients (17). The symptomatic patients showed longer dissection tendency compared with asymptomatic patients (20).

Ulcer-like projection (ULP) was firstly reported as one of the radiological findings of aortic dissection and explained as a localized collection of contrast material within the aortic wall (21). ULP has been considered as direct flow communication between TL and FL as well as might suggest a new intimal disruption or the intimal tear (22). The 35% (22/61) of patients with aortic ULP and thrombosed FL progressed to aortic rupture or enlargement (22). However, the distinct clinical and pathological significance of ULP in SISMAD has not been well investigated. Of the 24 Zerbib type III patients with ULP and thrombosed FL, complete remodeling was observed in 14 patients (58.3%) and progression to type II was confirmed in 10 patients (41.7%). Follow-up CTA indicated that no retrograde dissociation occurred from ULP toward SMA orifice. This study demonstrated that the distance between SMA orifice and ULP severely affected the radiological course after conservative management in type III SISMAD. ULP is always located at the convex curvature of the SMA, and the nearby ULP from the orifice of the SMA may be prone to suffer from the larger shearing forces. One conservatively treated patient type III has been reported to show a progressively dilated ULP, which was successfully treated with stent placement and coil packing (13). Hence, long-term CT follow-up are necessary if initial CT demonstrated a ULP on the patients with the thrombosed FL of the SMA.

Zerbib type IV patients of intramural hematoma (87.5%, 14/16) achieved complete remodeling due to hematoma absorption, which is strong evidence for the efficacy of conservative management. Two patients (12.5%, 2/16) with intramural hematoma encountered radiological progression to type II lesion with an entry and a patent “cul-de-sac” FL following the close follow-up. Intramural hematoma, known as the completely thrombosed FL, is characterized by the absence of intimal tear and direct flow communication between TL and FL. The extensive thrombus in the FL might contribute to the process of wall remodeling (10). Spontaneous isolated intramural hematoma was more likely to be angiographic improvement to complete remodeling due to hematoma absorption (23).

Anticoagulation has been advocated to prevent thrombosis of distal mesenteric arterial bed especially in SISMAD patients with occluded or severely compromised SMA blood flow. However, there are no consensuses on whether anticoagulation therapy should be routinely administered in patients with SISMAD. Anticoagulation can prevent false lumen thrombosis at SISMAD, thus promote further propagation of dissection. We did not appreciate a difference in outcome with use of anticoagulation or anti-platelet agents for SISMAD patients. A retrospective study enrolled 116 patients over the past 15 years and indicated that antithrombotic therapy offered no beneficial effects on either clinical or morphologic outcomes (9). No consensus has been reached with respect to anticoagulation therapy in the context of SISMAD management. A comparative study was needed to investigate the clinical and morphologic outcomes between anticoagulation use and no anticoagulation use.

### Limitations

This study has some limitations. First, it was a single-center, retrospective observational study with a limited number of patients. Limited by a retrospective study design, selection bias and confounding factors were unavoidable. Second, follow-up CT was performed at different time points in each patient, and some follow-up duration was less than 6 months. The natural history of SISMAD under conservative management should be observed over a longer period. Third, we did not investigate the effect of anticoagulant agents on clinical outcomes and morphologic changes of SISMAD. Randomized clinical studies with a large number of cases and long-term follow-up are needed to evaluate the benefit or potential risk of the anticoagulation therapy. Fourth, hypertension and smoking have been implicated as potential risk factors of SISMAD. Further studies would be necessary to clarify whether hypertension and smoking affect the remodeling of SISMAD.

## Conclusion

SISMAD might experience complete remodeling, partial remodeling, no change, dissection progression, type change after conservative management. SISMAD with a patent FL and a distal re-entry tends to be no change, whereas a thrombosed FL is the positive factor for complete remodeling. Partial remodeling, no change, and morphologic progression might occur in SISMAD with a patent “cul-de-sac” FL but no re-entry, in which the length of FL enhancement ≥ 40.3 millimeters is a strong predictor for dissection progression explained as the enlargement of a patent “cul-de-sac” FL. SISMAD with a thrombosed FL and ULP accomplishes complete remodeling or changes into a distinguish type with patent blinding-ending FL, in which the distance between SMA orifice and ULP ≤ millimeters 23.5 is an independent factor promoting the type change.

## Data Availability Statement

The raw data supporting the conclusions of this article will be made available by the authors, without undue reservation.

## Ethics Statement

Written informed consent was not obtained from the individual(s) for the publication of any potentially identifiable images or data included in this article.

## Author Contributions

ZY: collection or assembly of data and statistical analysis. SS and YY: collection or assembly of data and data analysis and interpretation. DL and KY: administrative support. QW: collection or assembly of data and provision of study material or patients. JW: financial support, conception and design, manuscript writing, and final approval of manuscript. All authors contributed to the article and approved the submitted version.

## Conflict of Interest

The authors declare that the research was conducted in the absence of any commercial or financial relationships that could be construed as a potential conflict of interest.

## Publisher’s Note

All claims expressed in this article are solely those of the authors and do not necessarily represent those of their affiliated organizations, or those of the publisher, the editors and the reviewers. Any product that may be evaluated in this article, or claim that may be made by its manufacturer, is not guaranteed or endorsed by the publisher.
